# Six-year follow-up of a survivor of cervical spine fracture and dislocation with oesophageal perforation following long scarf syndrome - a case report and literature review

**DOI:** 10.1186/s12891-020-03684-6

**Published:** 2020-10-14

**Authors:** Xiang Li, Fangyong Wang, Junwei Zhang, Yi Hong, Yong Yang

**Affiliations:** 1 Department of Orthopaedics, Beijing Friendship Hospital, Capital Medical University, No 95 Yongan Road Xicheng District, Beijing, 100050 China; 2 School of Rehabilitation Medicine, China Capital Medical University, Beijing, 100068 China; 3 Department of Spine and Spinal Cord Surgery, Beijing Boai Hospital, China Rehabilitation Research Center, No 10 North Jiaomen Road Fengtai District, Beijing, 100068 China

**Keywords:** Isadora duncan syndromeLong scarf syndromeCervical spine traumaOesophageal perforationConservative treatmentCase report

## Abstract

**Background:**

Accidental strangulation due to scarf getting caught in the wheels of a vehicle or machine was called “Isadora Duncan Syndrome” or “Long Scarf Syndrome”. Survival of concomitant fracture dislocation of cervical spine and oesophageal perforation following Long Scarf Syndrome was rarely described and medium-term follow-up for this lesion has not been reported.

**Case presentation:**

We present a 39-year-old female who suffered accidental strangulation caused by the scarf around her neck getting trapped in the wheels of the a vehicle and was referred to our hospital forty days post injury. The CT examination showed a fracture dislocation at C5/6 levels with complete dissociation of the supporting structures. She developed paravertebral abscesses, cutaneous fistulas and oesophageal perforation confirmed by oesophagoscope. The patient was treated conservatively because of poor general condition and inappropriate initial treatment. Halo-vest was used to immobilize the cervical spine. The oesophagus-cutaneous fistula was managed with enteral tube feeding and repeated local care. The patient survived despite such severe injury. Nine months after the injury, the oesophageal perforation closed spontaneously and fixed malunion of the cervical spine was achieved. Six-year follow-up demonstrated that the patient survived with complete C5 tetraplegia. Literature associated with this lesion was reviewed and factors contributing to the survival were discussed.

**Conclusions:**

Concomitant fracture dislocation of cervical spine and oesophageal perforation following Long Scarf Syndrome is extremely rare with high risk of mortality. Though surgical intervention is always necessary, the optimal management for this kind of lesion should be made on an individual basis through a multidisciplinary approach.

## Background

Accidental strangulation due to scarf getting entangled in the wheels of a vehicle or moving machine has been reported previously [1–6]. Accidents resulting from this mechanism of injury are called “Isadora Duncan Syndrome” or “Long Scarf”.

Syndrome [1, 5].The injury may range from contusion over the neck, transient loss of consciousness, cervical spine injury, cervical vascular injury, larynx fracture, oesophagus perforation and even death. Concomitant fracture dislocation of cervical spine and oesophageal perforation following Long Scarf Syndrome was rarely described [1]. To the best of our knowledge, six-year follow-up for this lesion has not been reported so far. The purpose of this article is to present such a patient who fortunately survived despite such severe injury and six-year follow-up was also achieved.

## Case presentation

**Fig. 1:**
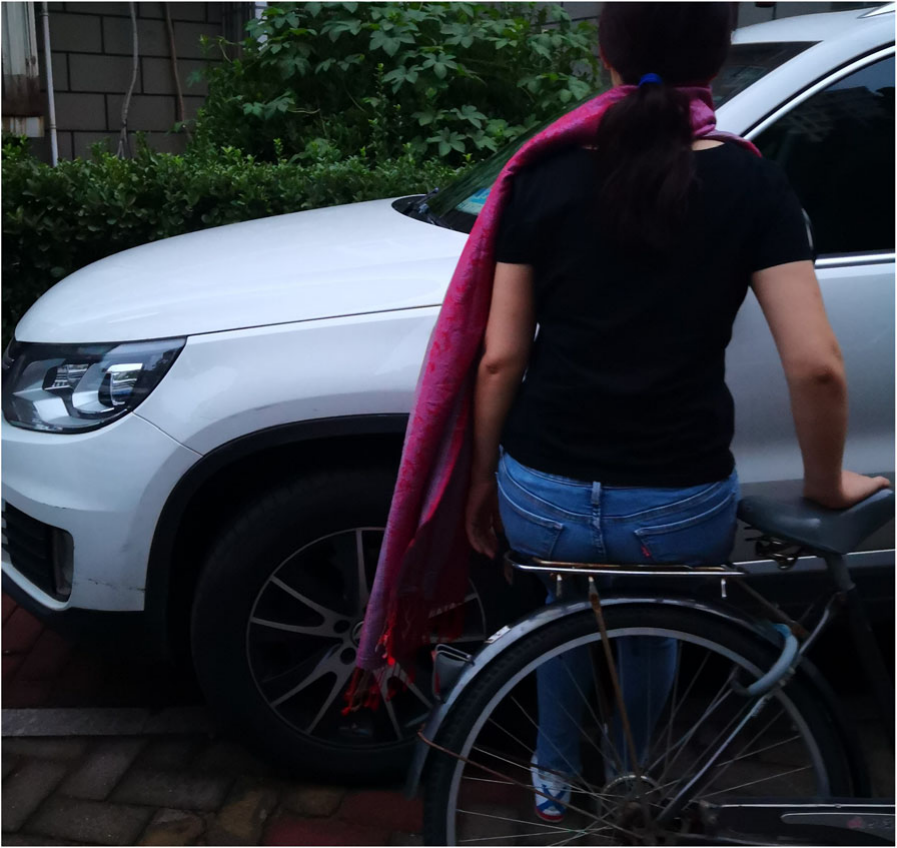
The reenacted image of the accident. A female with long scarf hanging by side exposed to danger of being caught in the wheels of a car

**Fig. 2:**
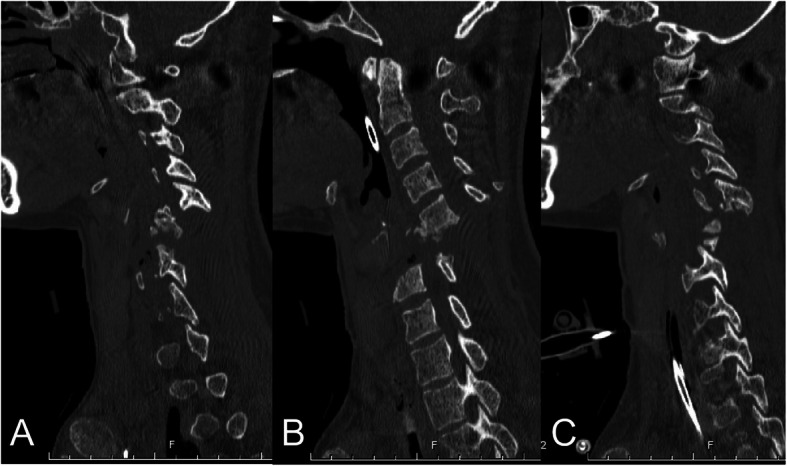
CT films showed fracture dislocation at C5-6 levels with significant widen at C5-6 intervertebral space

The patient was brought to the emergency department of our institution by ambulance. The medical records from the initial treated hospital showed the patient was a 39-year-old female. She was a pillion rider on the way to work. The long scarf she wore around her neck got caught in the wheels of a car and caused ligature effect across her neck (Fig. 1). She initially had a transient loss of consciousness. After receiving first aid at the scene, she was transferred to the nearest general hospital. CT examination revealed fracture dislocation at C5/6 levels and significant posterior displacement of C5 on C6. There was significant widening of the C5/6 intervertebral space which indicated complete dissociation of the supporting structure (Fig. 2). Associated injuries including pulmonary and splenic contusion were also revealed.

Emergency endotracheal intubation and assisted ventilation were performed due to progressive respiratory distress. Cervical collar was applied to immobilize the cervical spine. Associated pulmonary and splenic contusion were treated conservatively. Two weeks after injury, tracheostomy was performed to provide better tolerance and easier cleaning of the secretion. Meanwhile the subcutanous abscesses on the bilateral cervical region were noted and the bilateral wounds opened spontaneously. Local care was undertaken and no further treatment was administered per the medical records from the referring hospital. Forty days post injury, the patient was subsequently transferred to our hospital for further treatment.

Clinical examination on admission revealed a complete C5 tetraplegia which meant the patient could only shrug the shoulders and flex the elbow joints. Skin fistulas were noted on the bilateral cervical region discharging significant amount of pus. She was admitted to intensive care unit and artificial ventilation continued. Following a multidisciplinary consultation,, halo-vest immobilization was undertaken to prevent secondary spinal cord injury due to cervical spine instability. Esophagoscope examination showed that the trachea was intact, however a defect on the posterior wall of the cervical esophagus, which corresponded to the level of C5/6 injury was identified.

No radiological examination was performed because of the impossibility of suspension of assisted ventilation. Given the patient’s poor general condition, significant edematous changes of the esophagus wall and long time delay between the time of injury and the diagnosis, a decision to treat the oesophageal perforation non-operatively was made and consented by the patient’s husband.

A enteral tube was inserted to exclude the esophagus and enteral nutrition was given. The bilateral cervical wounds were irrigated twice a day with povidone-iodine fluid. Bacteriologic cultures disclosed positive for Pseudomonas aeruginosa and Klebsiella pneumoniae and intravenous antibiotic was given according to drug sensitivity test.

**Fig. 3:**
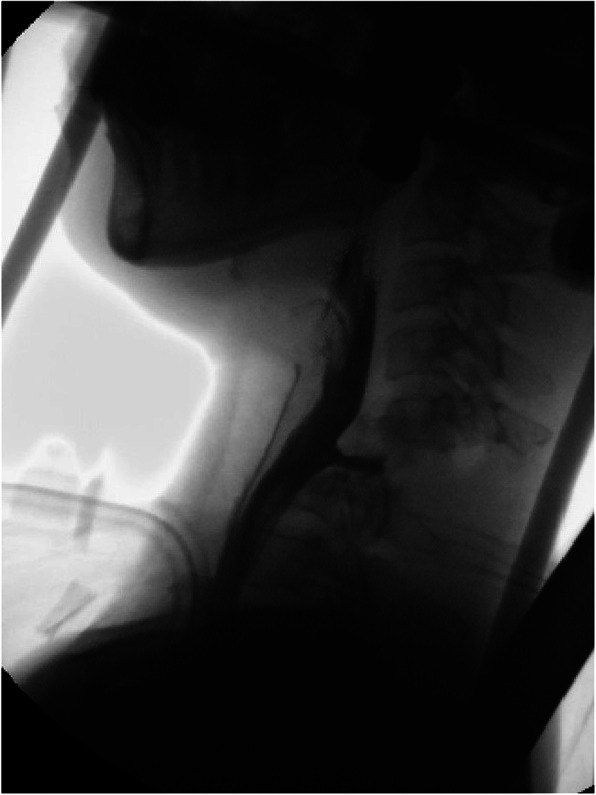
Oesophagography demonstrated the significant leakage of the contrast to the prevertebral space at the level of C5/6 injury

**Fig. 4:**
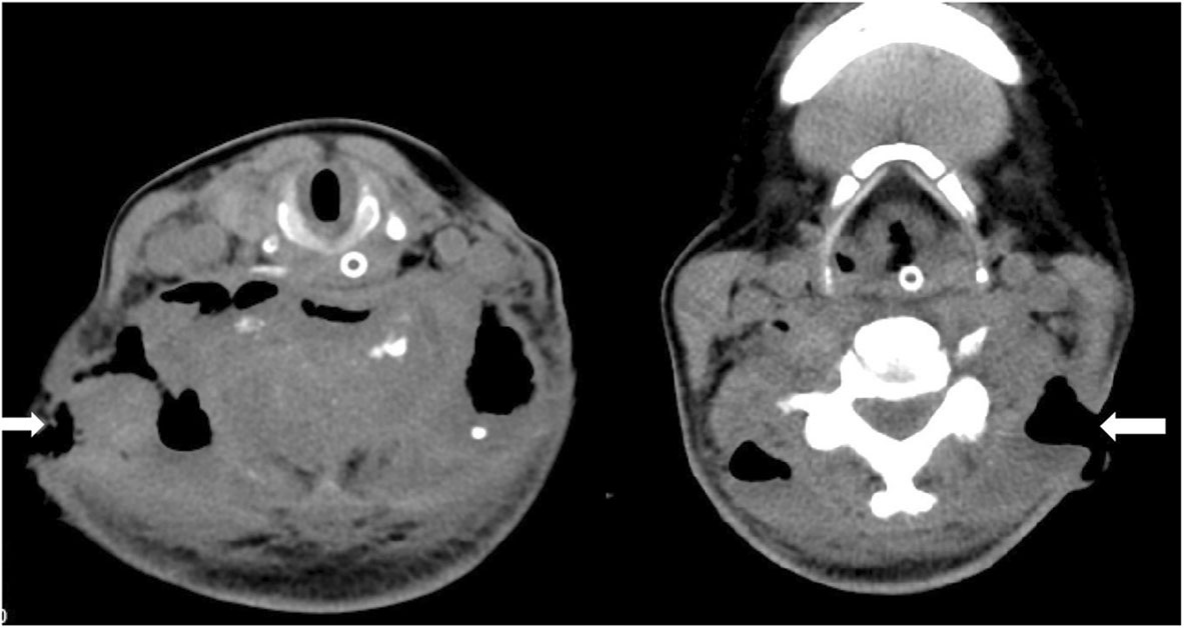
Cervical CT presented bilateral prevertebral abscesses, subcutaneous emphysema and skin fistulas (demonstrated by the arrows)

**Fig. 5:**
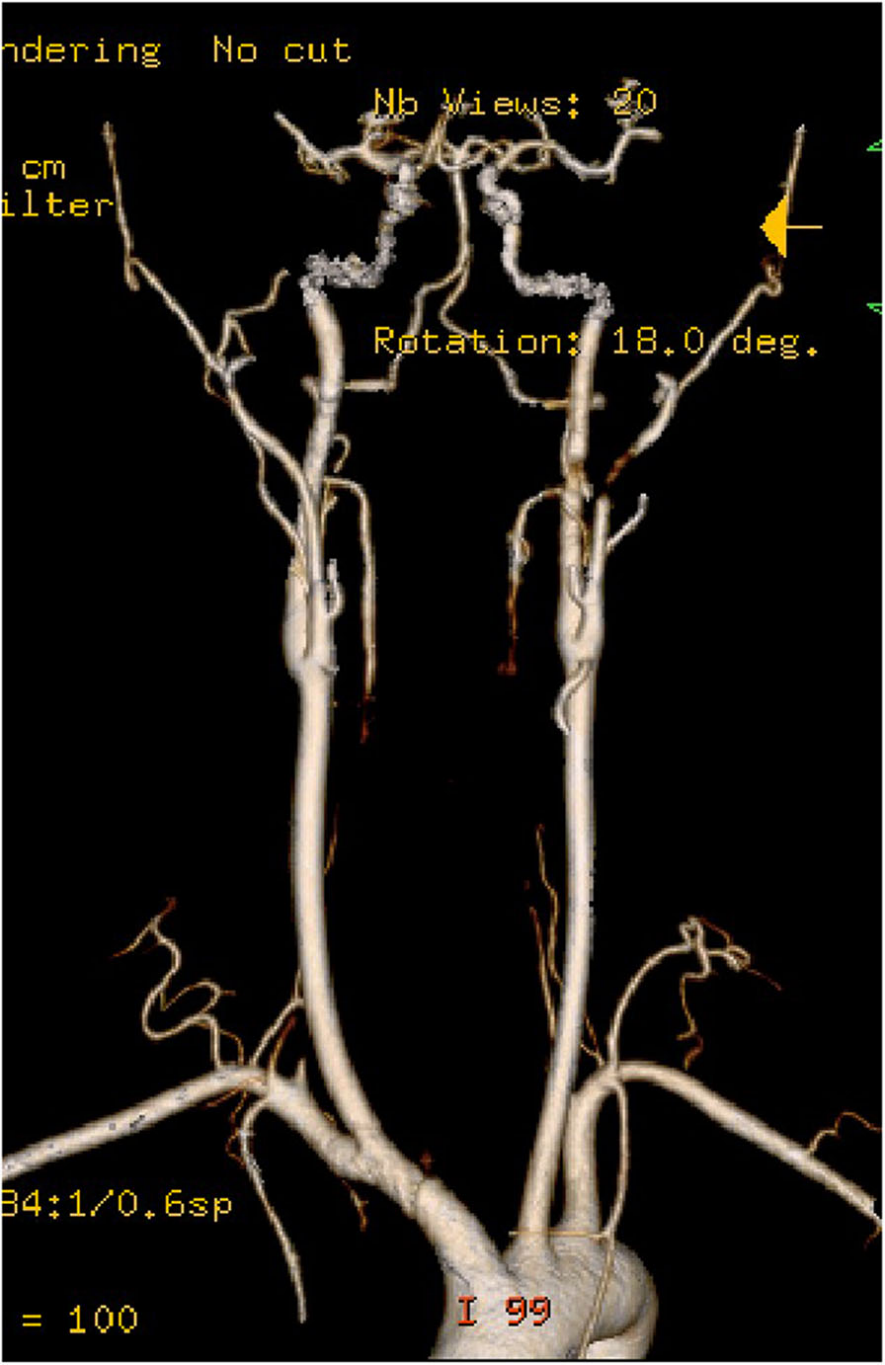
CTA demonstrated the narrowing of bilateral vertebral arteries consistent with the spinal injury levels

Three months post injury, the patient was successfully weaned off the ventilator which made it possible to undergo the requested radiological examinations. Oesophagography with water-soluble contrast swallow demonstrated the significant leakage of the contrast to the prevertebral space without extension to the mediastinum(Fig. 3). Cervical CT presented bilateral paravertebral abscesses, subcutaneous emphysema and skin fistulas (Fig. 4). Cervical CTA demonstrated the narrowing of bilateral vertebral arteries corresponding to the spinal injuries levels (Fig. 5), while the cerebellar CT was normal. The patient was transferred to the department of spine surgery and oesophagus exclusion and local care for cervical wound were continued.

**Fig. 6:**
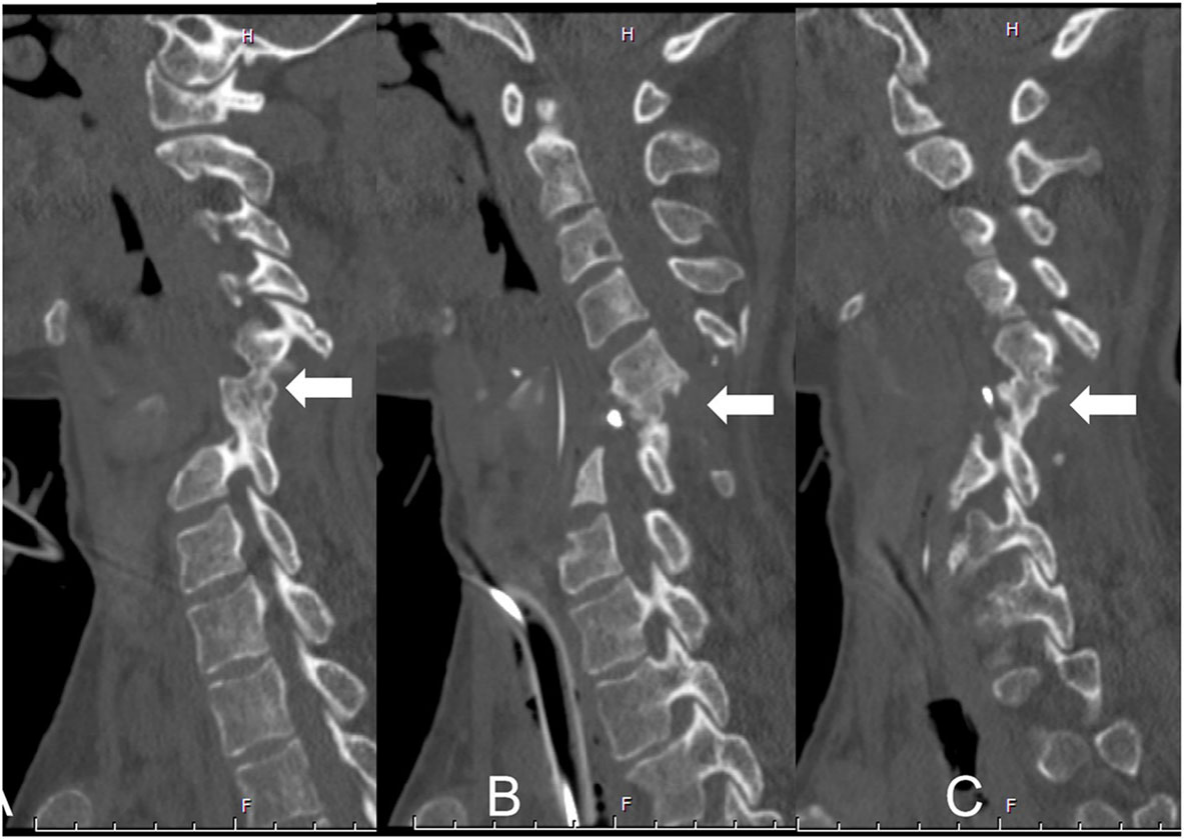
Cervical CT nine months post injury showed fixed malunion of the cervical spine (demonstrated by the arrows)

Nine months post injury, the cervical skin fistulas healed and oesophagography confirmed the healing of the oesophageal perforation. Cervical CT reported fixed malunion of the cervical spine (Fig. 6) but the dynamic radiographs were not achieved. The halo-vest brace and enteral tube were removed and oral feeding was allowed gradually. Eighteen months following injury, the tracheostomy tube was removed and the wound closed after a minor debridement and suture. The patient was then discharged home.

**Fig. 7:**
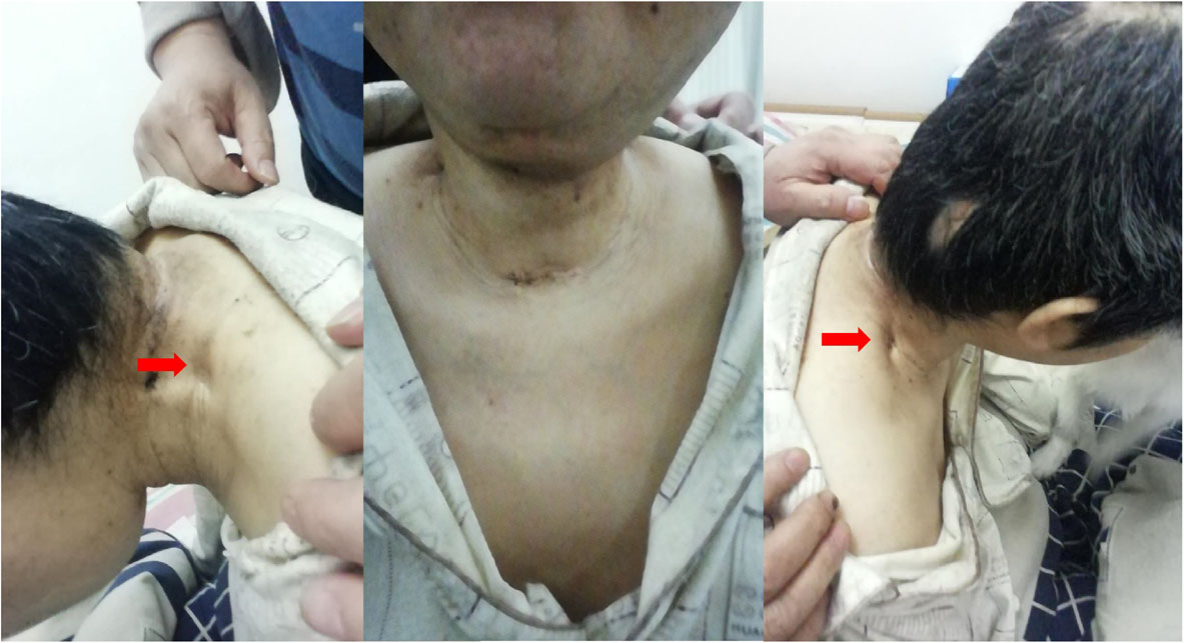
Photos taken 6 years after injury showed the scar of the site of oesophagus-skin fistulas (demonstrated by the arrows)

Six-year follow-up demonstrated that the patient survived with C5 complete spinal cord injury. She had achieved sitting balance and is independent in self-feeding and partial personal-hygiene such as face washing, tooth brushing and hair care with the aid of specialized equipment. The oesophagus-skin fistulas healed well (Fig. 7) and the patient reported no significant difficulty in swallowing. She presented with limited range of motion of cervical spine and moderate neck pain but refused to take further examination and treatment.

## Discussion and Conclusions

Accidental strangulation due to clothes worn around the neck getting caught in the wheels of a vehicle or moving machine has been documented, which is associated with high mortality [2–6].

The first reported case of death secondary to accidental strangulation was the world famous dancer Isadora Duncan, whose scarf got entangled in the wheel of a car and died at the scene. Postmortem examination showed she had a fractured larynx and carotid artery dissection [5]. Nowadays majority of this kind of accidents occur in the Indian subcontinent. Dupatta, a long scarf traditionally worn around the neck by Indian women when working exposes them to the danger of the free end getting trapped in belt driven powered machine such as crop thresher or tubewell, which can cause ligature effect across the neck [6]. Contemporarily, accidents resulting from the above mechanism of injury are more commonly called “Long Scarf Syndrome”.

Correlation between Long Scarf Syndrome and fracture dislocation of the cervical spine was rarely reported. Jain et al. [6, 7] reported 12 cases with cervical spine injury due to long scarf entanglement in machinery or wheels of vehicles. To the best of our knowledge, this is the largest series associated with cervical spinal injury secondary to Long Scarf Syndrome. Only two of these patients presented complete dislocation of cervical spine which was similar with the case presented. The other patients just presented subluxation of the cervical spine. In two patients with complete dislocation of cervical spine, one patient died three days after the injury while the other underwent combined anterior and posterior reconstruction of the cervical spine. The associated injuries such as oesophageal perforation or tracheal laceration were not mentioned in the article.

Cervical oesophageal perforations are rare but potentially life threatening injuries. About 1% of patients with cervical spinal cord injury may suffer associated perforation or laceration of the oesophagus, while forty percent of such lesion is directly due to the cervical spine fracture [7]. Different mechanisms have been proposed for the oesophageal injury: hyperextension and traction of the oesophagus; entrapment of the oesophageal wall between the separated vertebral bodies during spontaneous reduction; impingement of the oesophagus on the advanced degenerative cervical spine and the rare results of a hyperflexion injury [8, 9]. In our case, the long scarf was worn around the patient’s neck with a complete cycle. When the free end got trapped in the wheel of a car with high speed, it acted on the neck and applied a multi-directional torsion force across the neck. The post-injury cervical spine CT films indicated a hyperflexion combined with vertical distraction injury. The site of oesophageal perforation demonstrated by the oesophagography was consistent with the level of spinal injury. The oesophageal laceration most likely occurred while the oesophagus was overstretched on the anterior aspect of C5/6 level where the oesophagus is relatively immobile [10].

Our case is extremely rare because of the coexistence of fracture dislocation of cervical spine and oesophageal perforation following Long Scarf Syndrome and the fact that the patient fortunately survived despite such severe injury after conservative treatment. To our knowledge, only one case has been published on this kind of compounded injury in the literature so far. Ahmad et al. [4] reported a case who suffered cervical spine trauma, tracheal and oesophageal injuries caused by scarf tied around her neck getting caught in the wheel of go-kart. Trauma CT demonstrated C3/4 subluxation which was corresponding with flexion-distraction injury according to the films presented in the article. On the second day post injury, the transected cervical oesophagus and trachea were repaired. On the third day after the injury, reconstruction of the cervical spine was performed with posterior reduction, lateral mass screws fixation and fusion. The patient survived with C5 complete tetraplegia. Endoscopic examination performed three weeks post-injury showed the oesophageal defect had not healed, while spontaneous closure was expected and no further management was taken.

By contrast with the above case, our patient presented with fracture dislocation of the C5/6 levels with complete dissociation of the supporting structures rather than subluxation of the cervical spine, which suggested more severe high-energy injury. Surgical interventions including reconstruction of the cervical spine, exploration of the neck and surgical repair of the oesophageal injury is the preferred option for the majority of surgeons. It may be helpful to stabilize the cervical spine, control the infectious source and facilitate postoperative rehabilitation as soon as possible [7, 11]. However the long time delay from the time injury to diagnosis, poor general condition of the patient and significant edematous change of the oesophageal wall led us have to manage our case conservatively. After a long-term duration of repeated local care and immobilization of the cervical spine with halo-vest, the patient fortunately survived. Given the poor prognosis of the neurological function, the malunion of the cervical spine was acceptable. It is quite disappointing that the final follow-up radiological films were not performed therefore we could not evaluate whether a post-traumatic syringomyelia was developed or not. Six-year follow-up showed the oesophageal-cutaneous fistula healed well and the patient had no significant difficulty in swallowing.

The survival of our patient after such a severe injury could be attributed to several factors. Firstly, the neurological level of the cervical spinal cord injury was at C5, which might partially lower the compromise of the respiratory and cardiovascular function after spinal cord injury. Secondly, the infection of the paravertebral space was fortunately localized to the neck without extension to mediastinum, which lowered the risk of severe sepsis and made repeated local care possible to control the infection [11, 12]. Thirdly, though a halo-vest may restrict respiratory movement, it however provided solid immobilization of the cervical spine and made the position changing more easily. With the immobilization of halo-vest, the patient could sit either on the bed or wheelchair and undergo some rehabilitation programs, which is of great importance to prevent complications associated with long-term bed rest after spinal cord injury. All of above measures provided facilitation of the patient’s care and minimized the risk of mortality.

This is the first report of a six-year follow-up survival of cervical spine fracture and dislocation with oesophageal perforation following Long Scarf Syndrome. Poor general condition and inappropriate initial treatment led us have to treat this patient conservatively. The length of hospitalization and concomitant cost should also be taken into consideration. The optimal management for this kind of lesion should be made on an individual basis through a multidisciplinary approach.

## Data Availability

Not applicable.
